# Supplementary material for: Reproductive Stage Drought Tolerance in Wheat: Importance of Stomatal Conductance and Plant Growth Regulators
Source: Genes (Basel). 2021 Oct 29;12(11):1742. doi: 10.3390/genes12111742 (PMC8623834; doi:10.3390/genes12111742)
Supplement: Supplementary file 1 [file genes-12-01742-s001.zip › Supplementary Methods.pdf]

Phytohormone Quantification

Method

Sample extraction

- Approximately 100 mg tissues were weighed and transferred into a 2-ml lysing tube containing ceramic beads (MP Biomedical, Australia)
- 70% methanol supplied with 500 ng/ml of internal standards was added to each sample tube – 800 µl for leaf and 400 µl for spike tissues.
- Samples were homogenized using Precellys 24 tissue homogenizer (Bertin Technologies, France) for 3 cycles of 30 s at 6000 rpm with 90 s pause between the cycles. The extracts were centrifuged at 13000 rpm for 15 min.
- Supernatants were transferred into a LC-MS glass vial and subjected to LC-MS analysis.

LC-MS analysis

- 5 µl of samples was injected into the LC-MS machine under the following condition.
    - LC machine: 1200 series, Agilent Technologies
    - LC column: Zorbax Eclipse EDB-C18, 2.1 × 100 mm, 1.8 micron, Agilent Technologies
    - Column temperature: 45°C
    - Mobile phase A: water + 0.1% formic acid
    - Mobile phase B: acetonitrile + 0.1% formic acid
    - LC gradient program

At	%A	%B
0 min	80%	20%
2 min	80%	20%
3 min	50%	50%
12 min	5%	95%
16 min	5%	95%
17 min	80%	20%
23 min	80%	20%

- MS machine: Triple-Quad 6410, Agilent Technologies
- Gas temperature: 250°C
- Gas flow: 13 l/min
- Nebulizer: 55 psi
- Capillary voltage: 5500 V (positive mode) and 4500 V (negative mode)
- MRM transitions

Compound	Precursor ion (m/z)	Production (m/z)	Retention time (min)	Dwell time (ms)	Collision energy (eV)	Polarity
**Standards**						
Zeatin	220.1	136.1	0.93	200	14	Positive
IAA	176.1	130.1	3.83	200	10	Positive
CA	149.1	103.1	5.19	200	20	Positive
IBA	204.1	186.1	5.48	200	10	Positive
MeIAA	190.1	130.0	5.79	200	16	Positive
Brass	481.5	315.3	6.18	200	10	Positive
MeJA	225.0	151.0	7.14	200	10	Positive
GA3	345.1	143.1	2.03	200	24	Negative
ICA	160.1	116.1	2.86	200	10	Negative
SA	137.0	93.0	3.97	200	16	Negative
ABA	263.1	153.1	4.92	200	8	Negative
JA	209.1	59.0	5.55	200	8	Negative
GA4	331.2	213.0	5.93	200	30	Negative
JA-Ile	322.1	129.9	6.19	200	24	Negative
OPDA	291.0	164.9	8.10	200	20	Negative
**Internal standards**						
d_5-Zeatin	225.2	137.1	0.92	200	20	Positive
d_2-IAA	178.1	132.0	3.79	200	12	Positive
d_7-CA	156.1	109.0	5.16	200	22	Positive
d_4-SA	141.0	97.1	3.88	200	16	Negative
d_6-ABA	269.1	159.1	4.92	200	8	Negative
H_2JA	211.1	59.0	5.99	200	12	Negative

- Injection was performed in 3 technical replicates for each sample.
- Calibration curves were created from 4-8 dilutions of commercial standard. The curve is varied in a range of 10-5,000 ng/ml based on concentration of each phytohormone in the samples.
- Concentrations were measured by comparing relative peak area against the standard curve.

Result

Calibration curves

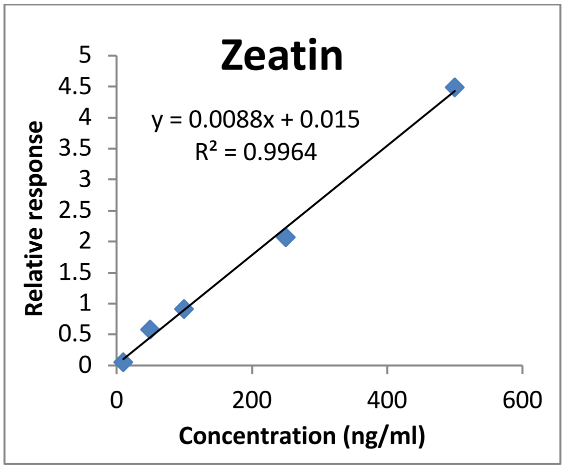

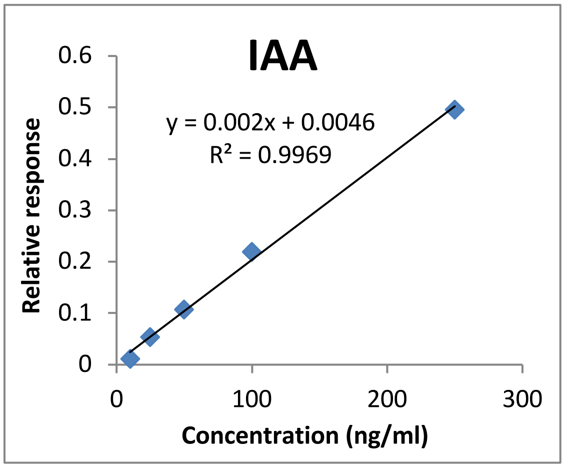

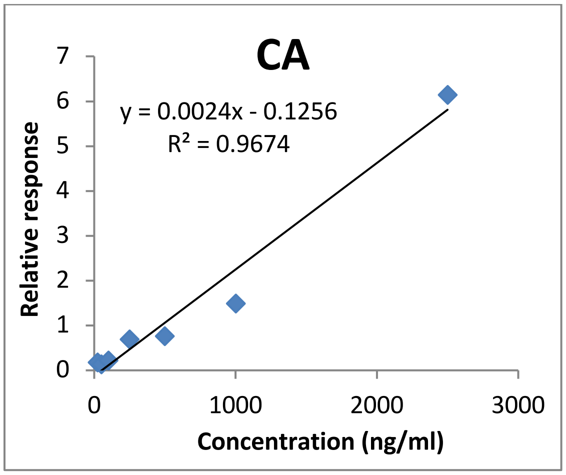

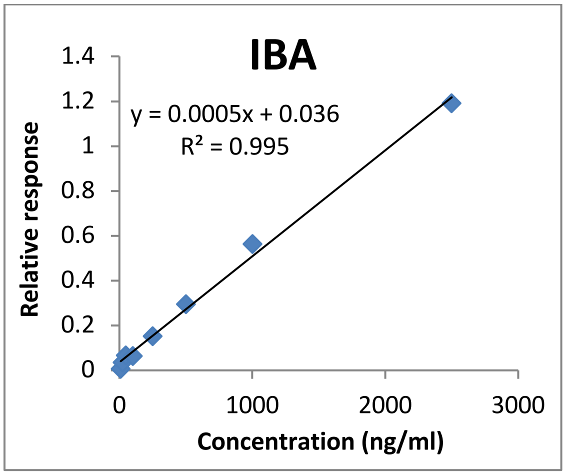

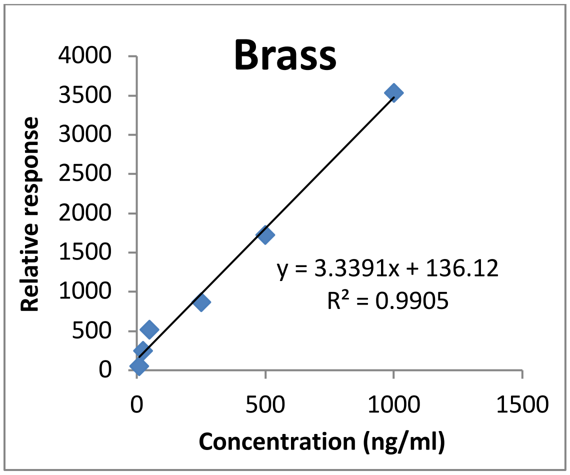
Brass
Relative response
Concentration (ng/ml)
y = 3.3391x + 136.12
R² = 0.9905
0
500
1000
1500
2000
2500
3000
3500
4000
0
500
1000
1500

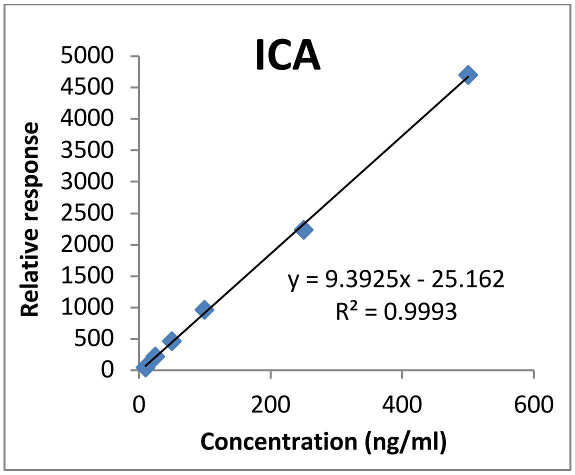
ICA
Relative response
Concentration (ng/ml)
y = 9.3925x - 25.162
R² = 0.9993
0
500
1000
1500
2000
2500
3000
3500
4000
4500
5000
0
200
400
600

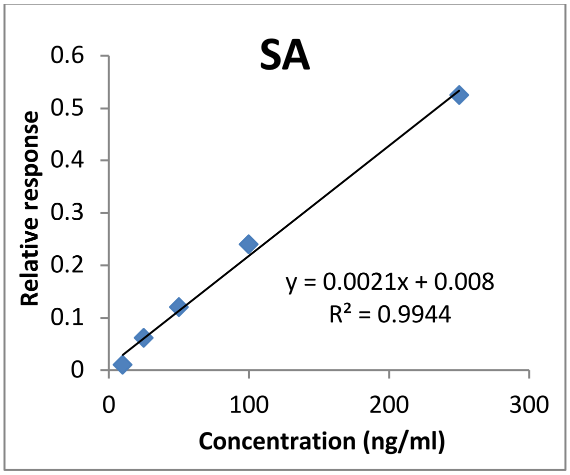
SA
Relative response
Concentration (ng/ml)
y = 0.0021x + 0.008
R² = 0.9944
0
0.1
0.2
0.3
0.4
0.5
0.6
0
100
200
300

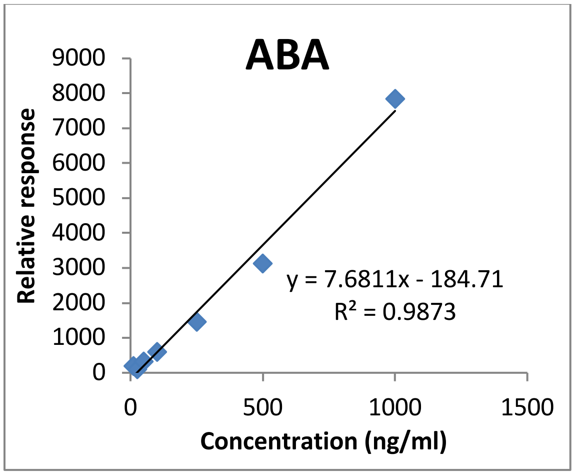
ABA
Relative response
Concentration (ng/ml)
y = 7.6811x - 184.71
R² = 0.9873
0
1000
2000
3000
4000
5000
6000
7000
8000
9000
0
500
1000
1500

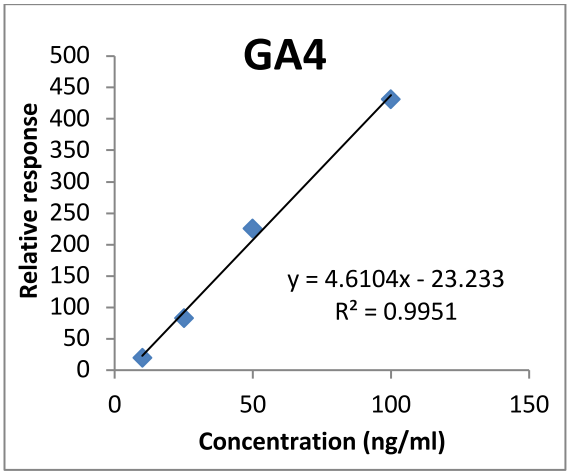
GA4
Relative response
Concentration (ng/ml)
y = 4.6104x - 23.233
R² = 0.9951
0
50
100
150
200
250
300
350
400
450
500
0
50
100
150

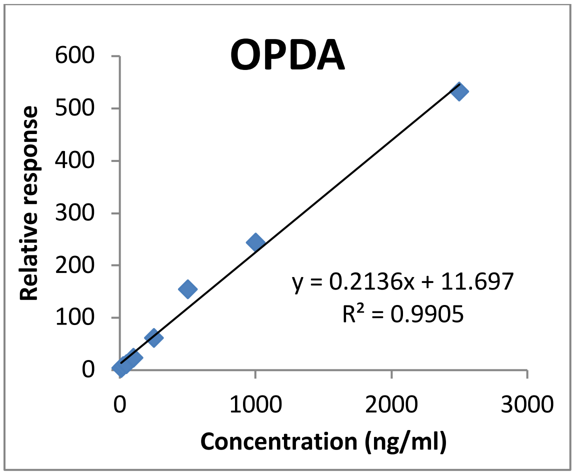
OPDA
Relative response
Concentration (ng/ml)
y = 0.2136x + 11.697
R² = 0.9905
0
100
200
300
400
500
600
0
1000
2000
3000

Example chromatograms

Zeatin

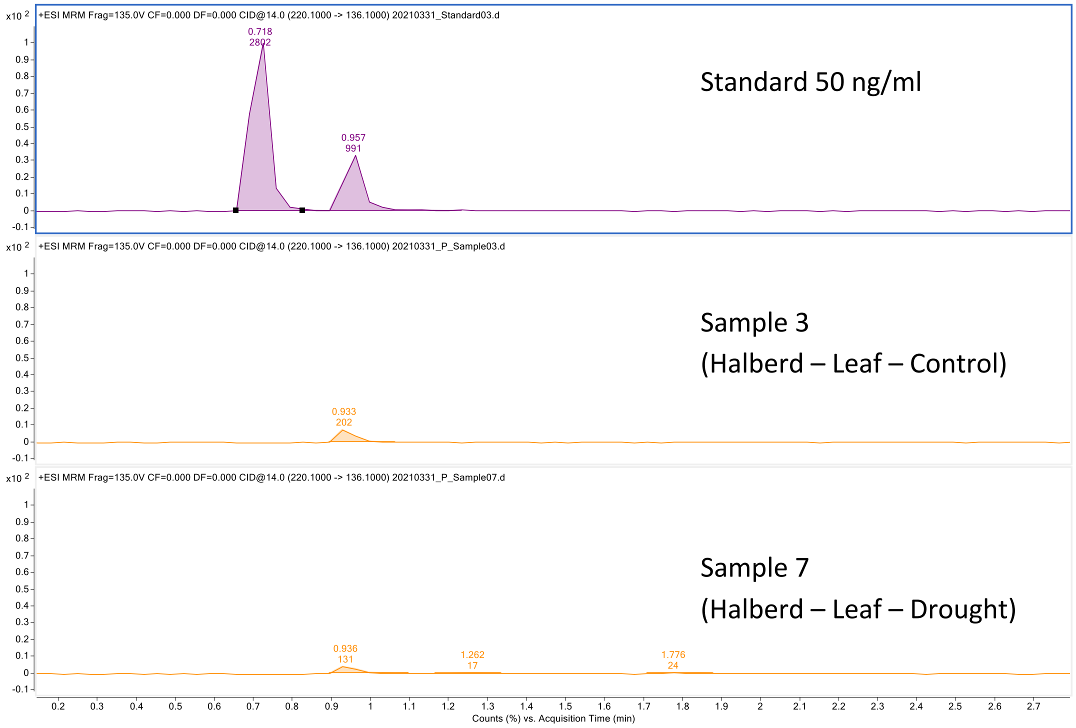

IAA

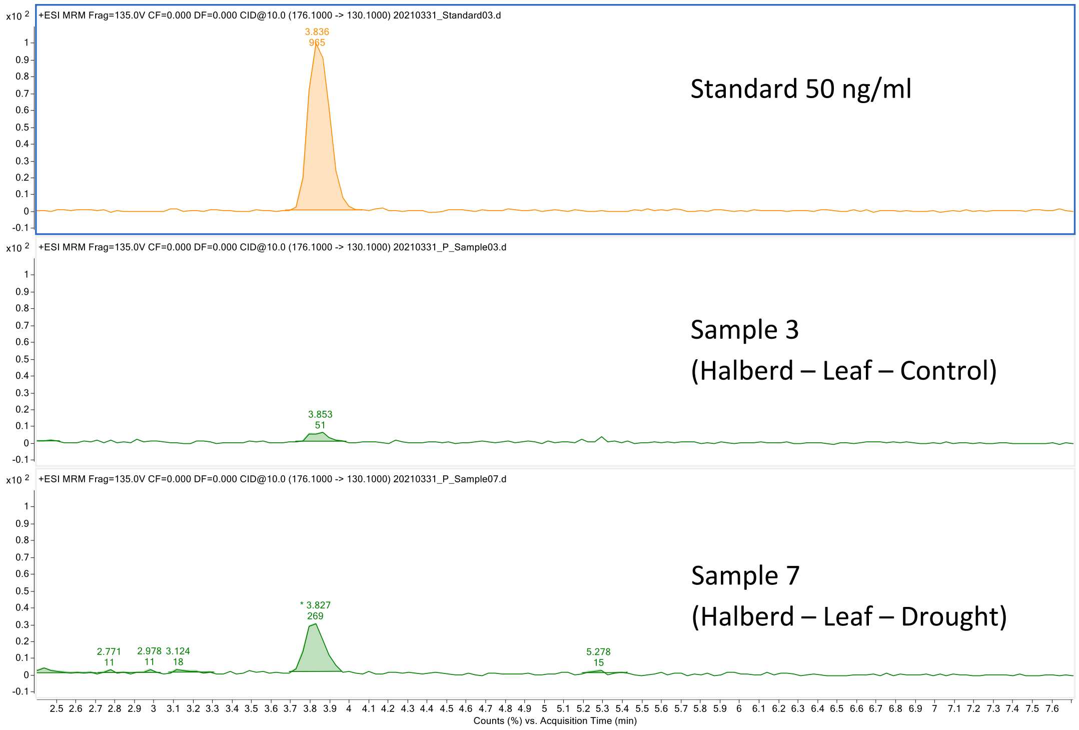

CA

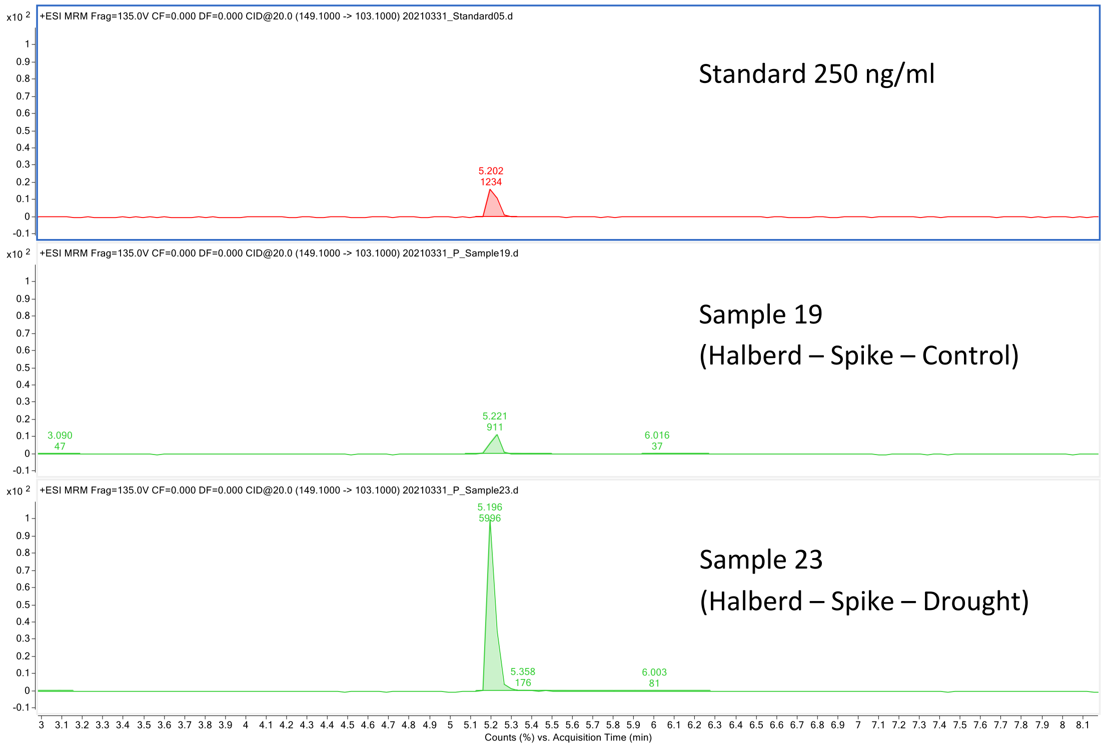

+ESI MRM Frag=135.0V CF=0.000 DF=0.000 CID@20.0 (149.1000 -> 103.1000) 20210331_Standard05.d
Standard 250 ng/ml
5.202
1234
+ESI MRM Frag=135.0V CF=0.000 DF=0.000 CID@20.0 (149.1000 -> 103.1000) 20210331_P_Sample19.d
Sample 19
(Halberd – Spike – Control)
3.090
47
5.221
911
6.016
37
+ESI MRM Frag=135.0V CF=0.000 DF=0.000 CID@20.0 (149.1000 -> 103.1000) 20210331_P_Sample23.d
Sample 23
(Halberd – Spike – Drought)
5.196
5996
5.358
176
6.003
81
Counts (%) vs. Acquisition Time (min)

IBA

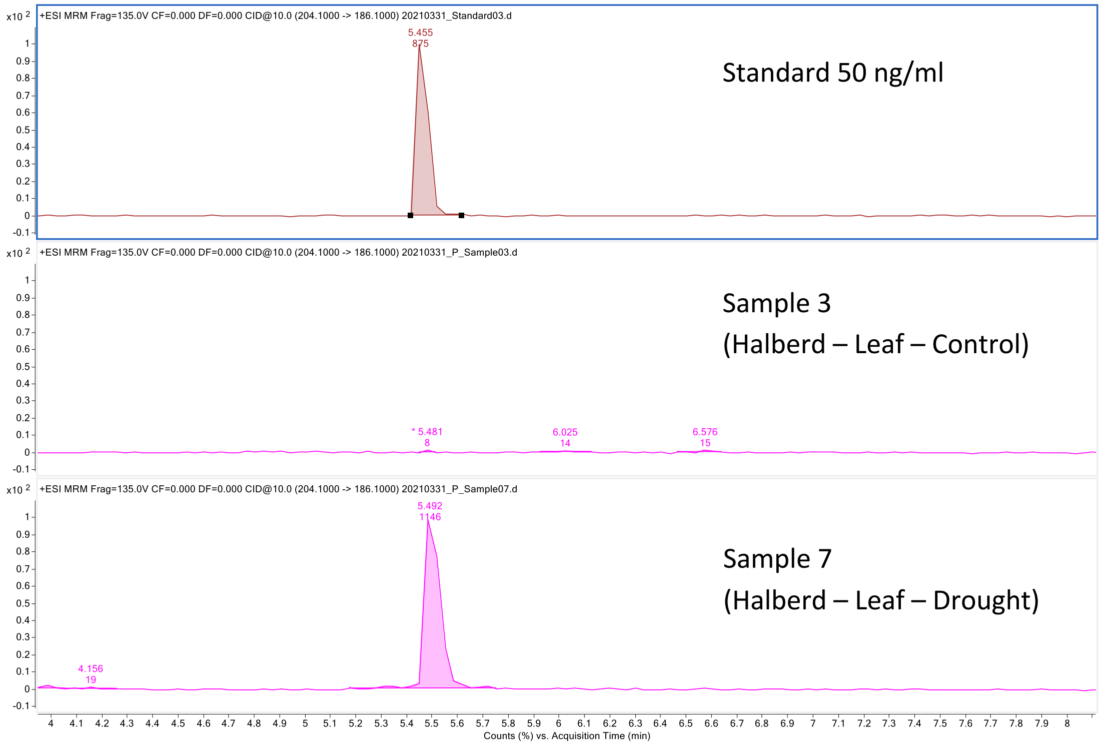

+ESI MRM Frag=135.0V CF=0.000 DF=0.000 CID@10.0 (204.1000 -> 186.1000) 20210331_Standard03.d
Standard 50 ng/ml
5.455
875
+ESI MRM Frag=135.0V CF=0.000 DF=0.000 CID@10.0 (204.1000 -> 186.1000) 20210331_P_Sample03.d
Sample 3
(Halberd – Leaf – Control)
* 5.481
8
6.025
14
6.576
15
+ESI MRM Frag=135.0V CF=0.000 DF=0.000 CID@10.0 (204.1000 -> 186.1000) 20210331_P_Sample07.d
Sample 7
(Halberd – Leaf – Drought)
5.492
1146
4.156
19
Counts (%) vs. Acquisition Time (min)

Brass

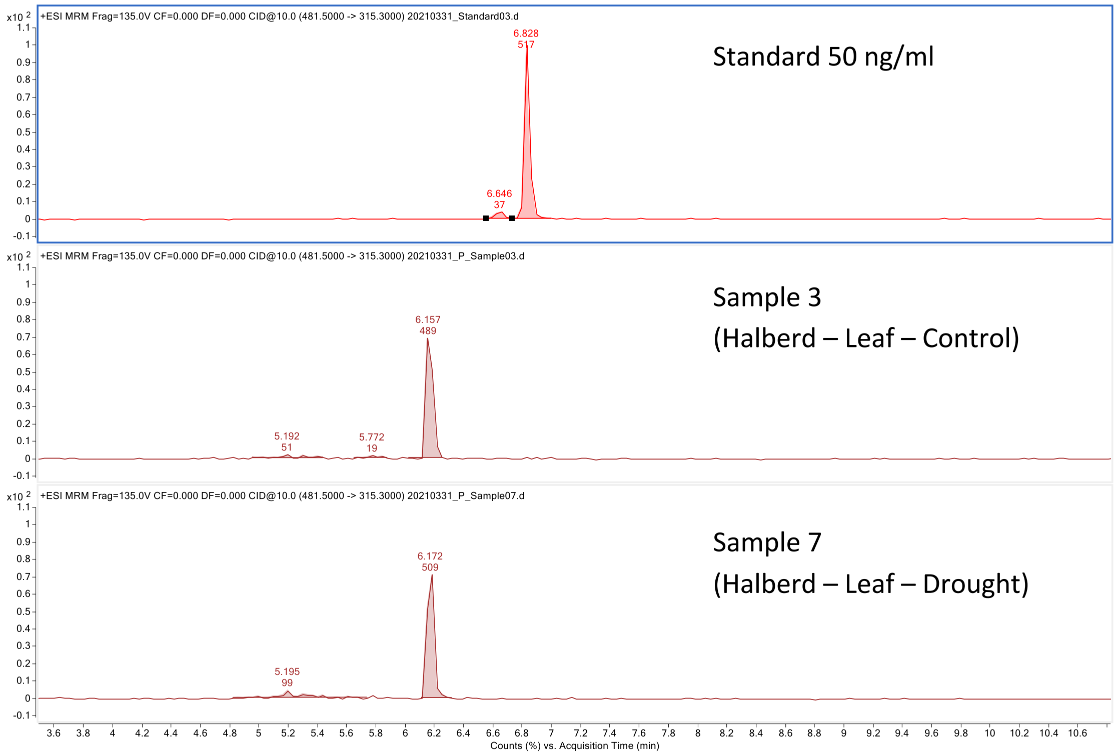
+ESI MRM Frag=135.0V CF=0.000 DF=0.000 CID@10.0 (481.5000 -> 315.3000) 20210331_Standard03.d
Standard 50 ng/ml
6.828
517
6.646
37
+ESI MRM Frag=135.0V CF=0.000 DF=0.000 CID@10.0 (481.5000 -> 315.3000) 20210331_P_Sample03.d
Sample 3
(Halberd – Leaf – Control)
6.157
489
5.192
51
5.772
19
+ESI MRM Frag=135.0V CF=0.000 DF=0.000 CID@10.0 (481.5000 -> 315.3000) 20210331_P_Sample07.d
Sample 7
(Halberd – Leaf – Drought)
6.172
509
5.195
99
Counts (%) vs. Acquisition Time (min)

ICA

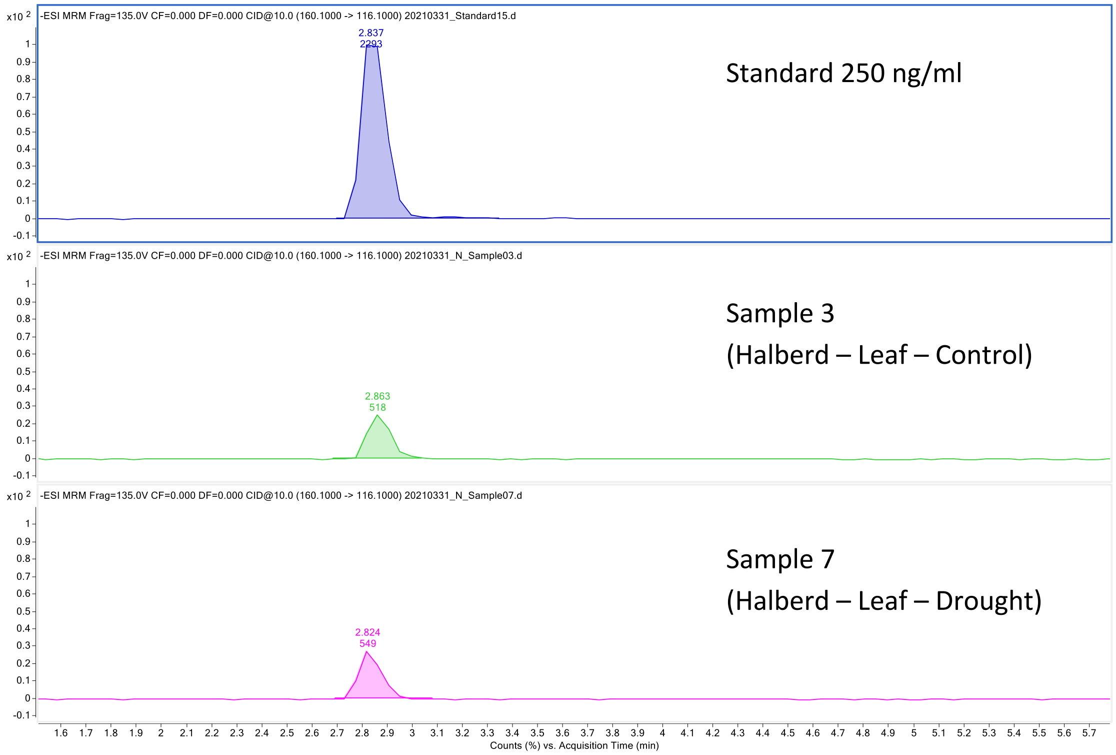
-ESI MRM Frag=135.0V CF=0.000 DF=0.000 CID@10.0 (160.1000 -> 116.1000) 20210331_Standard15.d
Standard 250 ng/ml
2.837
2293
-ESI MRM Frag=135.0V CF=0.000 DF=0.000 CID@10.0 (160.1000 -> 116.1000) 20210331_N_Sample03.d
Sample 3
(Halberd – Leaf – Control)
2.863
518
-ESI MRM Frag=135.0V CF=0.000 DF=0.000 CID@10.0 (160.1000 -> 116.1000) 20210331_N_Sample07.d
Sample 7
(Halberd – Leaf – Drought)
2.824
549
Counts (%) vs. Acquisition Time (min)

SA

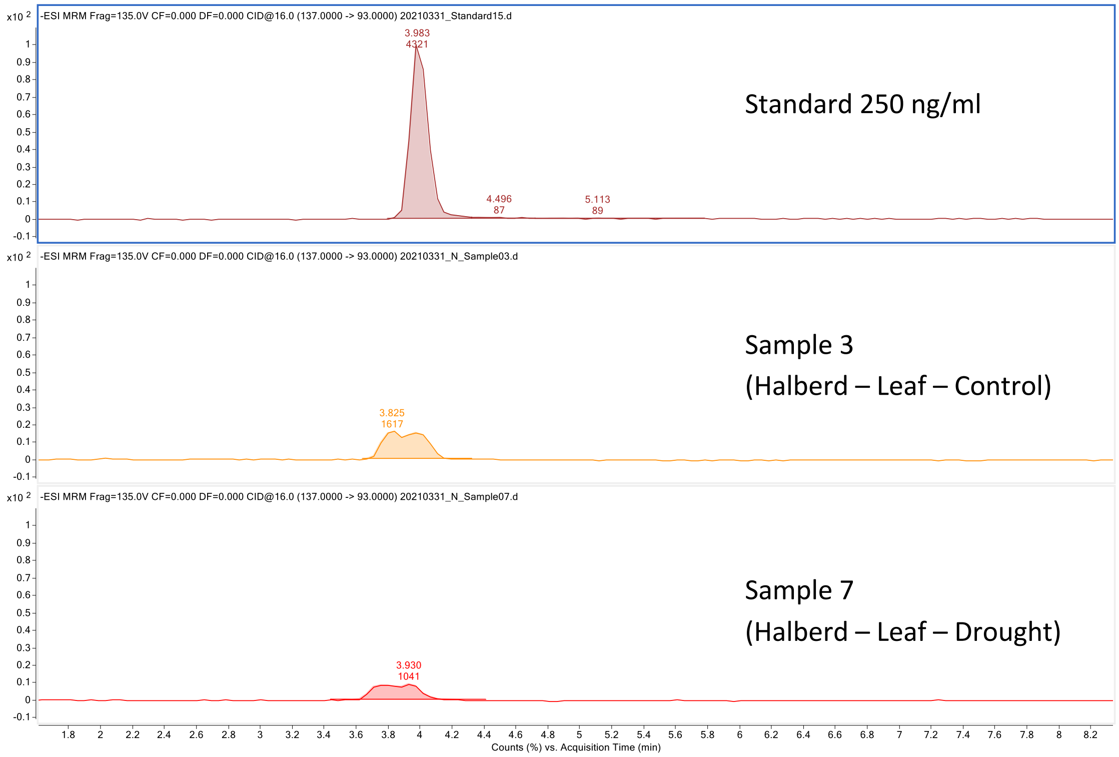

-ESI MRM Frag=135.0V CF=0.000 DF=0.000 CID@16.0 (137.0000 -> 93.0000) 20210331_Standard15.d
Standard 250 ng/ml
3.983
4321
4.496
87
5.113
89
-ESI MRM Frag=135.0V CF=0.000 DF=0.000 CID@16.0 (137.0000 -> 93.0000) 20210331_N_Sample03.d
Sample 3
(Halberd – Leaf – Control)
3.825
1617
-ESI MRM Frag=135.0V CF=0.000 DF=0.000 CID@16.0 (137.0000 -> 93.0000) 20210331_N_Sample07.d
Sample 7
(Halberd – Leaf – Drought)
3.930
1041
Counts (%) vs. Acquisition Time (min)

ABA

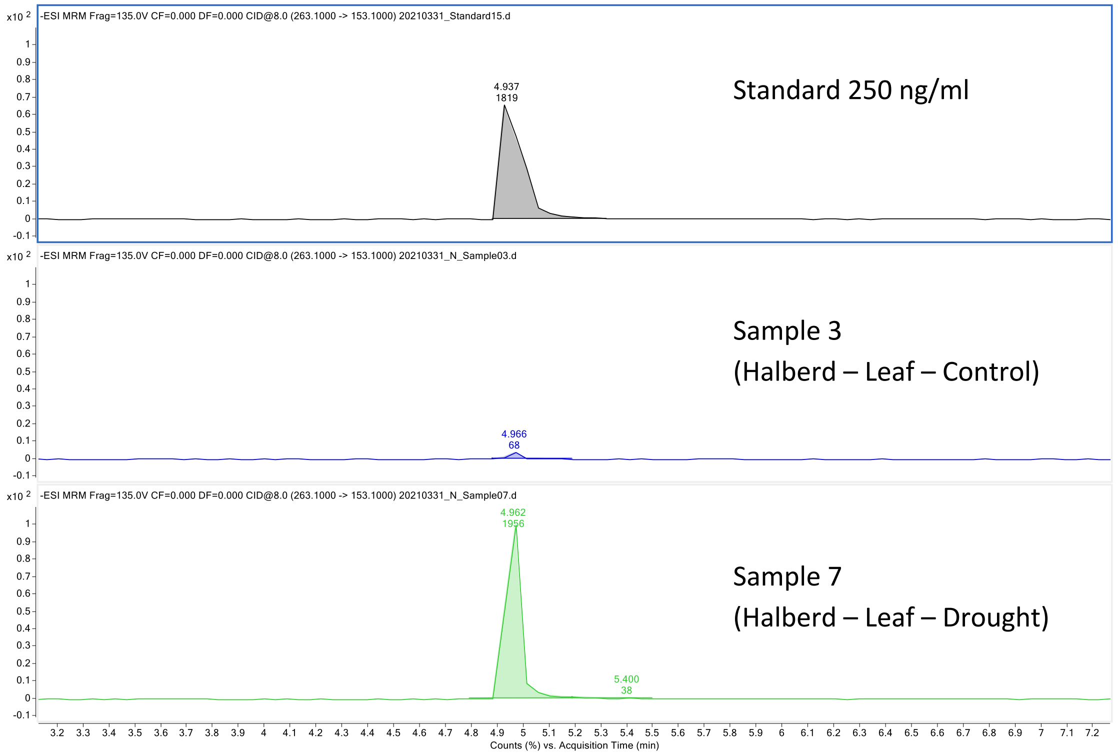

-ESI MRM Frag=135.0V CF=0.000 DF=0.000 CID@8.0 (263.1000 -> 153.1000) 20210331_Standard15.d
Standard 250 ng/ml
4.937
1819
-ESI MRM Frag=135.0V CF=0.000 DF=0.000 CID@8.0 (263.1000 -> 153.1000) 20210331_N_Sample03.d
Sample 3
(Halberd – Leaf – Control)
4.966
68
-ESI MRM Frag=135.0V CF=0.000 DF=0.000 CID@8.0 (263.1000 -> 153.1000) 20210331_N_Sample07.d
4.962
1956
Sample 7
(Halberd – Leaf – Drought)
5.400
38
Counts (%) vs. Acquisition Time (min)

JA-Ile

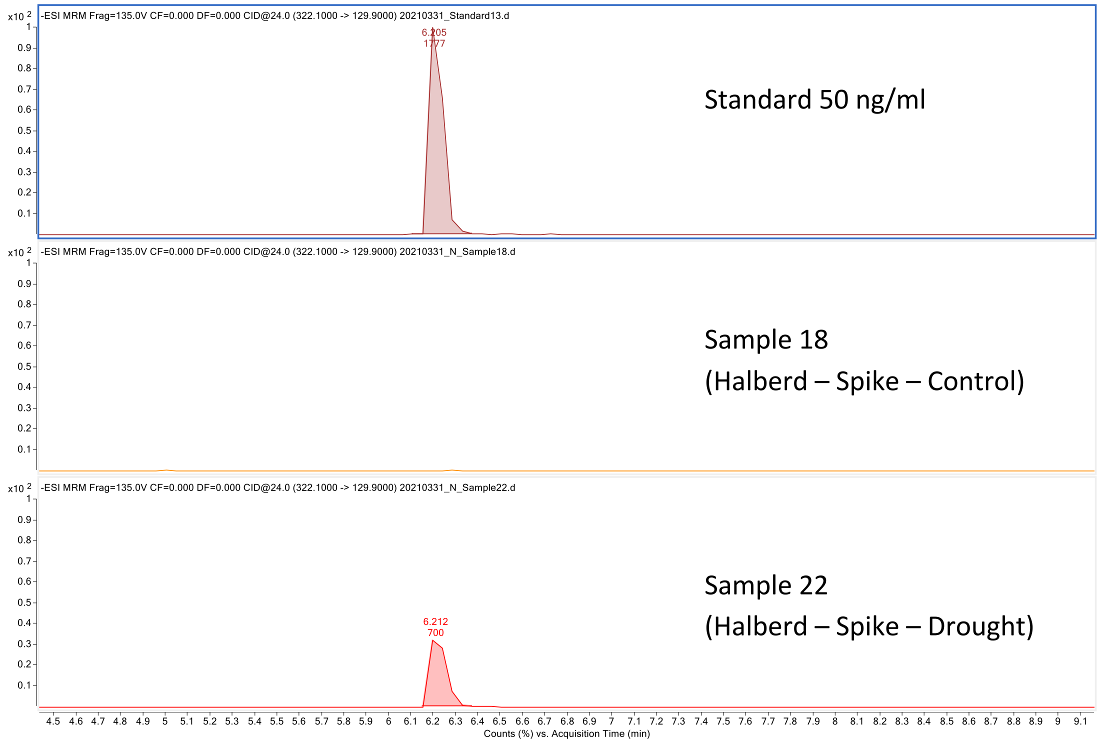

-ESI MRM Frag=135.0V CF=0.000 DF=0.000 CID@24.0 (322.1000 -> 129.9000) 20210331_Standard13.d
6.205
1777
Standard 50 ng/ml
-ESI MRM Frag=135.0V CF=0.000 DF=0.000 CID@24.0 (322.1000 -> 129.9000) 20210331_N_Sample18.d
Sample 18
(Halberd – Spike – Control)
-ESI MRM Frag=135.0V CF=0.000 DF=0.000 CID@24.0 (322.1000 -> 129.9000) 20210331_N_Sample22.d
6.212
700
Sample 22
(Halberd – Spike – Drought)
x10 2
Counts (%) vs. Acquisition Time (min)

OPDA

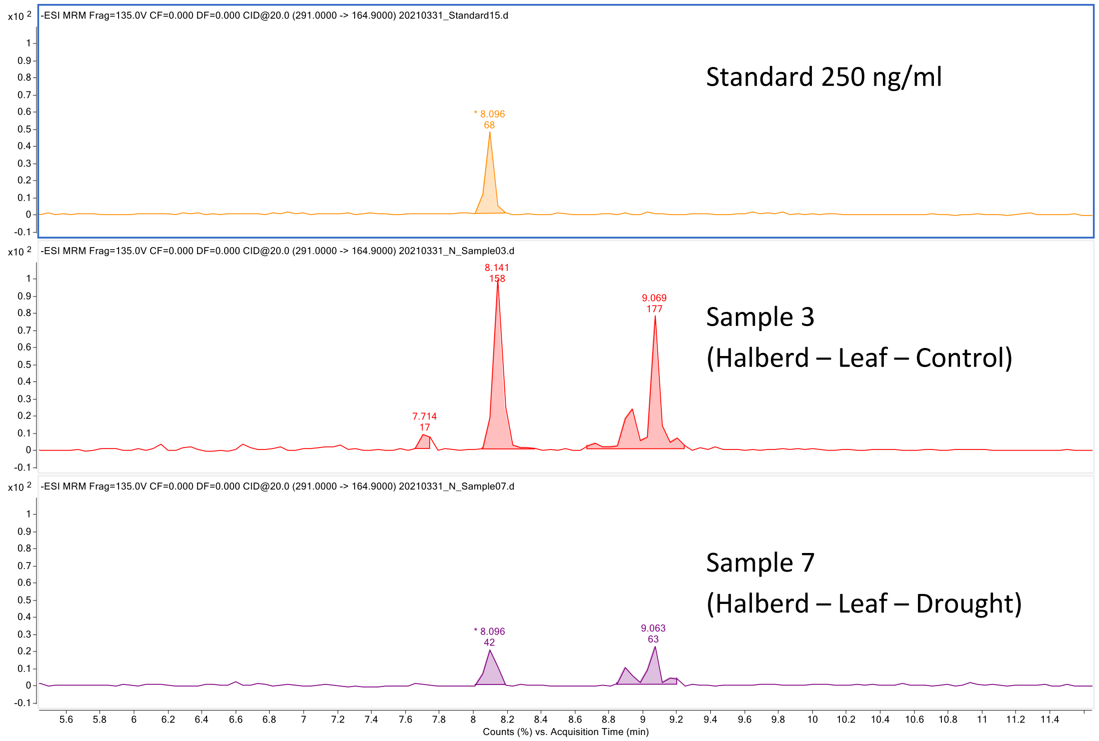

-ESI MRM Frag=135.0V CF=0.000 DF=0.000 CID@20.0 (291.0000 -> 164.9000) 20210331_Standard15.d
* 8.096
68
Standard 250 ng/ml
-ESI MRM Frag=135.0V CF=0.000 DF=0.000 CID@20.0 (291.0000 -> 164.9000) 20210331_N_Sample03.d
8.141
158
9.069
177
7.714
17
Sample 3
(Halberd – Leaf – Control)
-ESI MRM Frag=135.0V CF=0.000 DF=0.000 CID@20.0 (291.0000 -> 164.9000) 20210331_N_Sample07.d
* 8.096
42
9.063
63
Sample 7
(Halberd – Leaf – Drought)
x10 2
Counts (%) vs. Acquisition Time (min)
