# Supplementary material for: Reproductive Stage Drought Tolerance in Wheat: Importance of Stomatal Conductance and Plant Growth Regulators
Source: Genes (Basel). 2021 Oct 29;12(11):1742. doi: 10.3390/genes12111742 (PMC8623834; doi:10.3390/genes12111742)
Supplement: Supplementary file 1 [file genes-12-01742-s001.zip › Table S1.pdf]

Halberd Flag Leaf BP GO Enrichment					
GO.ID	Term	Annotated	Significant	Expected	classic
GO:0050794	regulation of cellular process	383	46	24.36	5.70E-06
GO:0050789	regulation of biological process	400	46	25.44	1.90E-05
GO:0006355	regulation of transcription, DNA-templat...	264	38	16.79	5.20E-07
GO:1903506	regulation of nucleic acid-templated tra...	264	38	16.79	5.20E-07
GO:2000112	regulation of cellular macromolecule bio...	272	38	17.3	1.10E-06
GO:0009790	embryo development	15	7	0.95	1.60E-05
GO:0006032	chitin catabolic process	22	7	1.4	0.00028
GO:0006869	lipid transport	20	6	1.27	0.00111
GO:0019684	photosynthesis, light reaction	5	5	0.32	9.90E-07
GO:0006086	acetyl-CoA biosynthetic process from pyr...	3	3	0.19	0.00025
GO:0006094	gluconeogenesis	3	3	0.19	0.00025
GO:0006099	tricarboxylic acid cycle	3	3	0.19	0.00025
GO:0006561	proline biosynthetic process	3	3	0.19	0.00025
GO:0006816	calcium ion transport	4	3	0.25	0.00097
GO:0006415	translational termination	5	3	0.32	0.0023
GO:0009269	response to desiccation	6	3	0.38	0.00438
GO:0006183	GTP biosynthetic process	8	3	0.51	0.01116
GO:0006228	UTP biosynthetic process	8	3	0.51	0.01116
GO:0015696	ammonium transport	8	3	0.51	0.01116
GO:0006536	glutamate metabolic process	11	3	0.7	0.02855
GO:0006813	potassium ion transport	13	3	0.83	0.04507
GO:0015743	malate transport	3	2	0.19	0.01156
GO:0006730	one-carbon metabolic process	6	2	0.38	0.05091

Cranbrook Flag Leaf BP GO Enrichment					
GO.ID	Term	Annotated	Significant	Expected	classic
GO:0019538	protein metabolic process	514	316	248.09	2.00E-11
GO:0010467	gene expression	413	273	199.34	2.10E-15
GO:0006796	phosphate-containing compound metabolic ...	413	257	199.34	4.90E-10
GO:0016070	RNA metabolic process	337	212	162.66	6.20E-09
GO:0006468	protein phosphorylation	298	182	143.84	1.80E-06
GO:0006355	regulation of transcription, DNA-templat...	264	167	127.43	1.90E-07
GO:0043043	peptide biosynthetic process	100	76	48.27	7.70E-09
GO:0006633	fatty acid biosynthetic process	85	49	41.03	0.0497
GO:0015672	monovalent inorganic cation transport	31	24	14.96	0.00084
GO:0006559	L-phenylalanine catabolic process	32	24	15.45	0.00182
GO:0072528	pyrimidine-containing compound biosynthe...	19	14	9.17	0.02206
GO:0046856	phosphatidylinositol dephosphorylation	13	12	6.27	0.00113
GO:0016192	vesicle-mediated transport	17	12	8.21	0.05353
GO:0006563	L-serine metabolic process	15	11	7.24	0.04451
GO:0006081	cellular aldehyde metabolic process	8	8	3.86	0.00291
GO:0006544	glycine metabolic process	8	7	3.86	0.02803
GO:0006656	phosphatidylcholine biosynthetic process	6	6	2.9	0.01257
GO:0009107	lipoate biosynthetic process	6	6	2.9	0.01257
GO:0009269	response to desiccation	6	6	2.9	0.01257
GO:0042128	nitrate assimilation	6	6	2.9	0.01257
GO:0006006	glucose metabolic process	5	5	2.41	0.0261
GO:0006012	galactose metabolic process	5	5	2.41	0.0261
GO:0007205	protein kinase C-activating G protein-co...	5	5	2.41	0.0261
GO:0009101	glycoprotein biosynthetic process	5	5	2.41	0.0261
GO:0006353	DNA-templated transcription, termination	4	4	1.93	0.05415
GO:0006680	glucosylceramide catabolic process	4	4	1.93	0.05415
GO:0015914	phospholipid transport	4	4	1.93	0.05415
GO:0032957	inositol trisphosphate metabolic process	4	4	1.93	0.05415
GO:0042372	phylloquinone biosynthetic process	4	4	1.93	0.05415
GO:0046168	glycerol-3-phosphate catabolic process	4	4	1.93	0.05415
