# Supplementary material for: Reproductive Stage Drought Tolerance in Wheat: Importance of Stomatal Conductance and Plant Growth Regulators
Source: Genes (Basel). 2021 Oct 29;12(11):1742. doi: 10.3390/genes12111742 (PMC8623834; doi:10.3390/genes12111742)
Supplement: Supplementary file 1 [file genes-12-01742-s001.zip › Table S2.pdf]

Halberd Spike BP GO Enrichment						Cranbrook Spike BP GO Enrichment					
**GO.ID**	**Term**	**Annotated**	**Significant**	**Expected**	**classic**	**GO.ID**	**Term**	**Annotated**	**Significant**	**Expected**	**classic**
GO:0050896	response to stimulus	372	221	167.73	1.90E-09	GO:0051179	localization	451	260	230.41	0.00137
GO:0005975	carbohydrate metabolic process	185	107	83.41	0.00021	GO:0051234	establishment of localization	449	258	229.39	0.00186
GO:0008610	lipid biosynthetic process	99	59	44.64	0.00222	GO:0006810	transport	449	258	229.39	0.00186
GO:0007017	microtubule-based process	60	49	27.05	4.50E-09	GO:0015979	photosynthesis	55	52	28.1	1.50E-12
GO:0007018	microtubule-based movement	59	48	26.6	8.30E-09	GO:0042592	homeostatic process	39	31	19.92	0.00022
GO:0009057	macromolecule catabolic process	65	47	29.31	6.40E-06	GO:0006820	anion transport	40	28	20.44	0.01146
GO:0009733	response to auxin	58	41	26.15	6.10E-05	GO:0045454	cell redox homeostasis	34	26	17.37	0.00208
GO:0006073	cellular glucan metabolic process	51	35	22.99	0.00053	GO:0033014	tetrapyrrole biosynthetic process	23	23	11.75	1.80E-07
GO:0007275	multicellular organism development	45	33	20.29	0.0001	GO:0006032	chitin catabolic process	22	19	11.24	0.00058
GO:0042546	cell wall biogenesis	46	27	20.74	0.04294	GO:0006418	tRNA aminoacylation for protein translat...	26	19	13.28	0.01862
GO:0034655	nucleobase-containing compound catabol...	25	18	11.27	0.00579	GO:0046148	pigment biosynthetic process	23	18	11.75	0.00692
GO:0006032	chitin catabolic process	22	17	9.92	0.00213	GO:0006814	sodium ion transport	16	16	8.17	2.10E-05
GO:0006260	DNA replication	23	17	10.37	0.00474	GO:0006364	rRNA processing	15	14	7.66	0.00063
GO:0000160	phosphorelay signal transduction system	19	13	8.57	0.03435	GO:0046856	phosphatidylinositol dephosphorylation	13	12	6.64	0.00213
GO:0007049	cell cycle	12	12	5.41	6.90E-05	GO:0016226	iron-sulfur cluster assembly	12	11	6.13	0.00389
GO:0009690	cytokinin metabolic process	15	12	6.76	0.00633	GO:0009082	branched-chain amino acid biosynthetic p...	10	10	5.11	0.00119
GO:0006308	DNA catabolic process	16	12	7.21	0.01497	GO:0006096	glycolytic process	7	7	3.58	0.00902
GO:0033875	ribonucleoside bisphosphate metabolic pr...	11	11	4.96	0.00015	GO:1901663	quinone biosynthetic process	6	6	3.07	0.01769
GO:0034032	purine nucleoside bisphosphate metabolic...	11	11	4.96	0.00015	GO:0006006	glucose metabolic process	5	5	2.55	0.03469
GO:0009269	response to desiccation	6	6	2.71	0.00835	GO:0006817	phosphate ion transport	5	5	2.55	0.03469
GO:0015969	guanosine tetraphosphate metabolic proce...	6	6	2.71	0.00835	GO:0007205	protein kinase C-activating G protein-co...	5	5	2.55	0.03469
GO:0006002	fructose 6-phosphate metabolic process	5	5	2.25	0.01855	GO:0046836	glycolipid transport	5	5	2.55	0.03469
GO:0006275	regulation of DNA replication	5	5	2.25	0.01855						
GO:0007205	protein kinase C-activating G protein-co...	5	5	2.25	0.01855						
GO:0006635	fatty acid beta-oxidation	4	4	1.8	0.04122						
